# Supporting data for senary refractory high-entropy alloy Cr*_x_*MoNbTaVW

**DOI:** 10.1016/j.dib.2015.10.027

**Published:** 2015-11-02

**Authors:** B. Zhang, M.C. Gao, Y. Zhang, S.M. Guo

**Affiliations:** a Louisiana State University, Baton Rouge, LA 70803, USA; b National Energy Technology Laboratory, Albany, OR 97321, USA; c AECOM, P.O. Box 1959, Albany, OR 97321, USA

## Abstract

This data article is related to the research paper entitled “senary refractory high-entropy alloy Cr*_x_*MoNbTaVW [1]”. In this data article, the pseudo-binary Cr-MoNbTaVW phase diagram is presented to show the impact of Cr content to the senary Cr-MoNbTaVW alloy system; the sub-lattice site fractions are presented to show the disordered property of the Cr-MoNbTaVW BCC structures; the equilibrium and Scheil solidification results with the actual sample elemental compositions are presented to show the thermodynamic information of the melted/solidified Cr*_x_*MoNbTaVW samples; and the raw EDS scan data of the arc-melted Cr*_x_*MoNbTaVW samples are also provided.

**Specifications table**

**Table:** 

Subject area	*Materials*
More specific subject area	*High entropy alloy (HEA)*
Type of data	*Table, figure*
How data was acquired	*EDS area scan, ThermoCalc Calculations*
Data format	*Analyzed*
Experimental factors	*The HEA samples were prepared using arc-melting of metallic powders. For material characterizations, the samples were sectioned and polished.*
Experimental features	*The EDS area scan was conducted and the ThermoCalc Calculations were performed based on the EDS measured compositions.*
Data source location	*Baton Rouge, Louisiana, USA*
Data accessibility	*The data are included in this article.*

**Value of the data**

• A full range pseudo-binary phase diagram of Cr*_x_*MoNbTaVW is provided, which can be used for future comprehensive studies on the Cr*_x_*MoNbTaVW high entropy alloy system.
• The sub-lattice composition data can guide researchers on alloy microstructure analyses.
• The data provide information on the thermodynamic behavior of Cr*_x_*MoNbTaVW HEA samples.

## 1 Data

Compared with the traditional single-principal-element alloys, high entropy alloys (HEA) have five or more principal metallic elements at near equal molar ratios and a simple phase crystal structure [2]. Based on the reported quinary MoNbTaVW alloy system [3] and Ti containing TiMoNbTaVW system [4], Cr is incorporated into the MoNbTaVW system to form a senary refractory HEA Cr*_x_*MoNbTaVW.

**Fig. 1:**
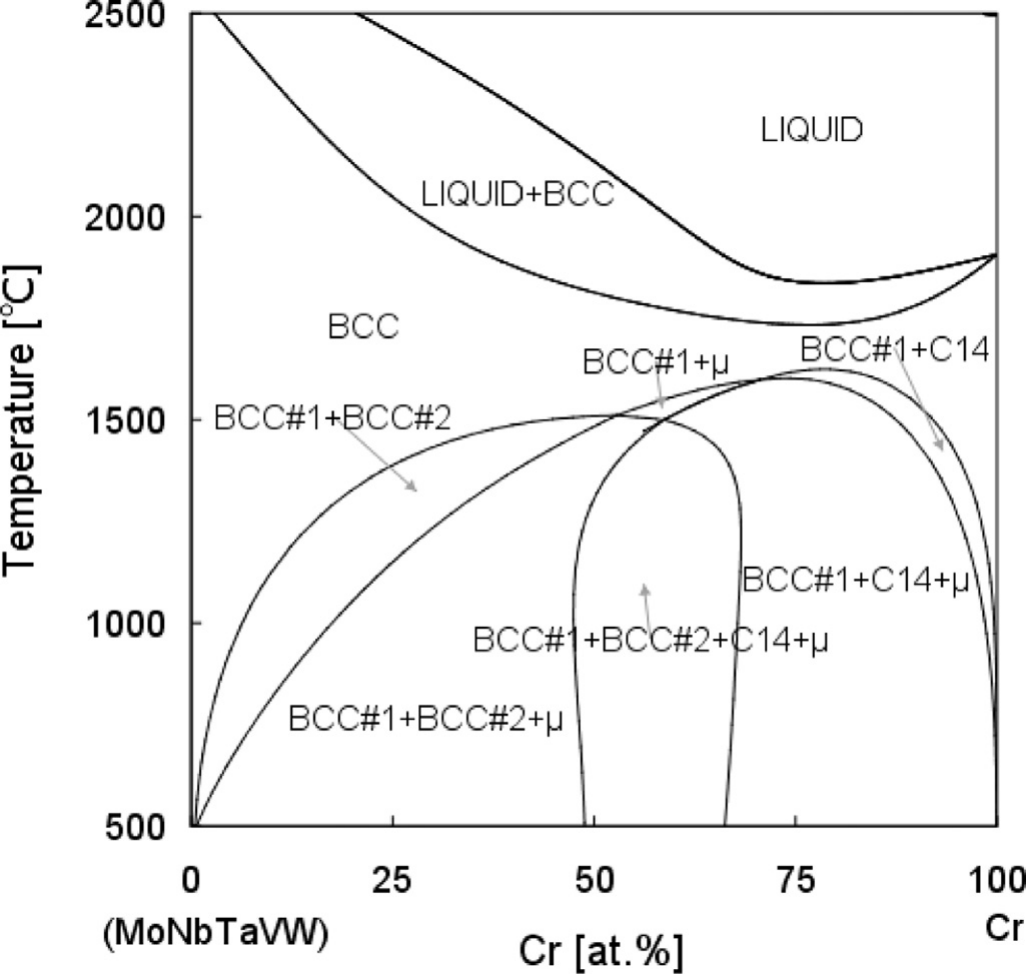
Calculated pseudo-binary phase diagram of Cr*_x_*MoNbTaVW.

To examine the effect of Cr concentration to the Cr_x_MoNbTaVW HEA system, quasi-binary phase diagram with the variation of Cr composition is presented in Fig. 1. As laves phase, a common intermetallic phase, exists in the Cr alloyed refractory alloys [5], the quasi-binary phase diagram can be used to guide the formation of single phased HEAs.

**Fig. 2:**
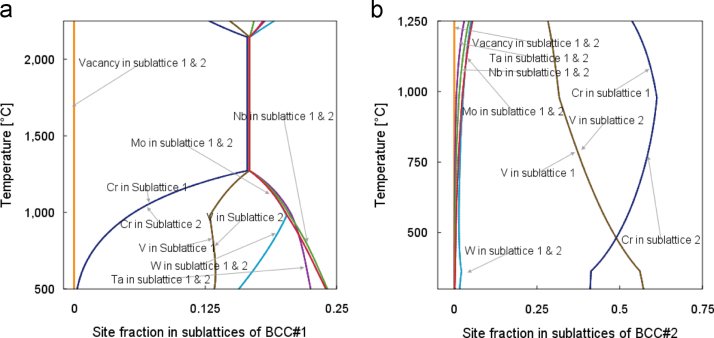
Sub-lattices 1 and 2 compositions of (a) BCC#1 and (b) BCC#2 in Cr_1_MoNbTaVW.

The composition data for two sub-lattices of BCC are calculated. The BCC lattice contains two interpenetrating simple cubic sub-lattices, one sub-lattice consisting of the cubes’ corners, and the other sub-lattice consisting of the cubes’ centers. In the ordered state, since certain atoms appear at certain spatial positions, the hypothetically divided sub-lattices 1 and 2 will be occupied by two different combinations of elements. That causes the elemental site occupancies of sub-lattices 1 and 2 differ to each other. In contrast, under a completely disordered state, a site can be occupied by an atom of any type, thus the probability for a given site containing an atom of a given type is equal to the probabilities for the other sites [6]. In Thermo-Calc^TM^ calculations, when the site occupancies of the two sub-lattices are the same, the structure is considered to be disordered [7]. Fig. 2 shows the site occupancy fractions are identical for both sub-lattices #1 and #2.

The initial Thermo-Calc^TM^ calculations for Cr*_x_*MoNbTaVW were based on the designed Cr contents (*x*=0.5, *x*=1.0, *x*=2.0) [1]. For experimental validations, samples for the targeted Cr ratios were synthesized and experimentally characterized. Although the compositions in the feedstock were based on the designed Cr contents (*x*=0.5, *x*=1.0, *x*=2.0), due to the fact that the boiling temperature of Cr is even less than the melting temperature of W, the loss of Cr during the arc melting process is intense. This excess vaporization causes the deviation of the bulk compositions from the targeted ratios. After the actual bulk compositions of three samples were determined through the EDS area scans, the CALPHAD calculation of the solidification process was re-performed using the measured EDS compositions. The CALPHAD simulations using these true compositions are presented in this data paper.

## 2 Experimental design, materials and methods

The Thermo-Calc^TM^ software was used to perform the CALPHAD calculations. In the Console mode of Thermo-Calc^TM^, after initialization, the thermodynamic database TCNI7 [7] was employed to cover all edge binaries and most available ternaries of the Cr–Mo–Nb–Ta–V–W system. Then the elements Cr, Mo, Nb, Ta, V and W were defined and all the phases were restored for exhaustive analysis. After defining the system, the POLY3 module was used for the phase diagram calculation. The initial conditions include temperature, pressure, and the equivalence properties for all elements besides Cr. The temperature and the Cr composition were set as variables and the searching ranges were also assigned. The POST module of Thermo-Calc^TM^ was used to plot the phase diagram after the mapping was completed, as shown in Fig. 1. In the associated research article [1], the range of Cr content was narrower, for batter matching of the experimentally tested Cr content range.

The site fractions of sub-lattices 1 and 2 of each BCC structure were plotted based on Thermo-Calc^TM^ simulations. After confirming the TCNI7 database, all six elements of Cr, Mo, Nb, Ta, V and W were defined and all the relevant phases were restored. Then the POLY3 module of the Thermo-Calc^TM^ was activated for the property diagram calculations. For the initial conditions, the ambient temperature and pressure were set and the mole fractions of all the elements were set to be 16.67%. The equilibrium single point calculation was initiated to determine a starting point for the step loop. The temperature range and increment were then set for the rest of step calculations. After the completion of step calculations, the site fractions of BCC#1 and BCC#2 phases were plotted in the POST module (Fig. 2(a) and (b)).

For experimental measurements of sample compositions, after sectioning an arc-melted HEA sample, the exposed surface of the ingot was subsequently ground by # 240, #400, #600, and #800 SiC sandpapers in sequence, before wet-polishing with 1, 0.3 and 0.05 µm Al_2_O_3_ suspensions. Field-emission scanning electron microscope FE-SEM (FEI, Quanta 3DFEG) equipped with BSE and EDS detectors was used to characterize the chemical compositions of the samples along the cross-sections. The EDS area scan was performed with a relatively low magnification (600×) and long live scan time (200 s) to obtain the element ratios.

**Fig. 3:**
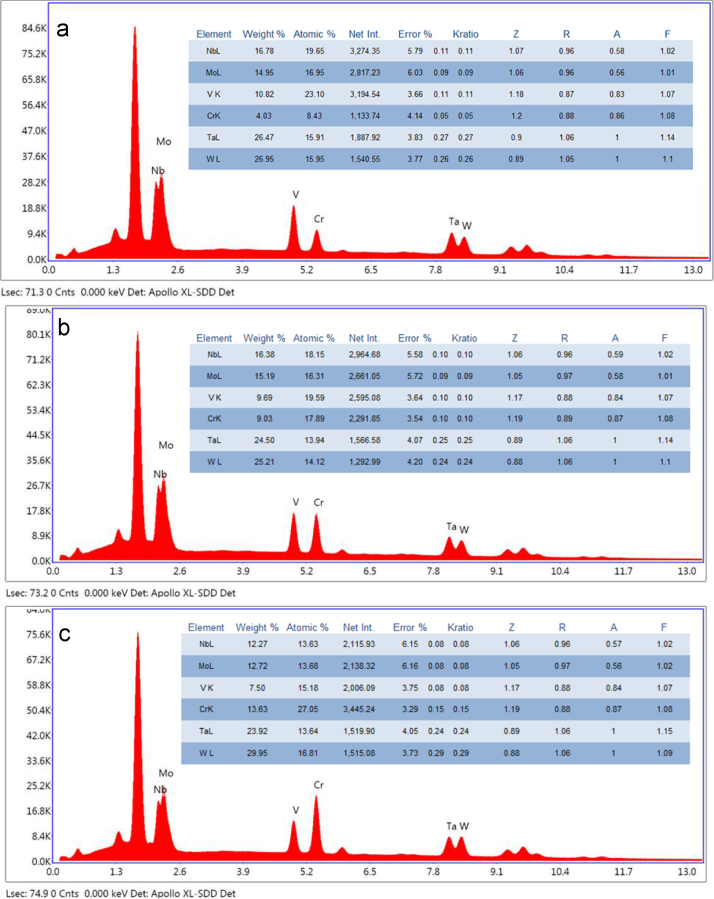
EDS bulk composition analysis results for Cr*_x_*MoNbTaVW HEAs: (a) *x*=0.5, (b) *x*=1.0 and (c) *x*=2.0.

The EDS measured elemental ratios, Fig. 3, were regarded as the bulk compositions and were used to re-perform the CALPHAD solidification calculations. Two different solidification models were used for all three cases: the equilibrium solidification and the Scheil–Gulliver solidification [8], [9].

**Fig. 4:**
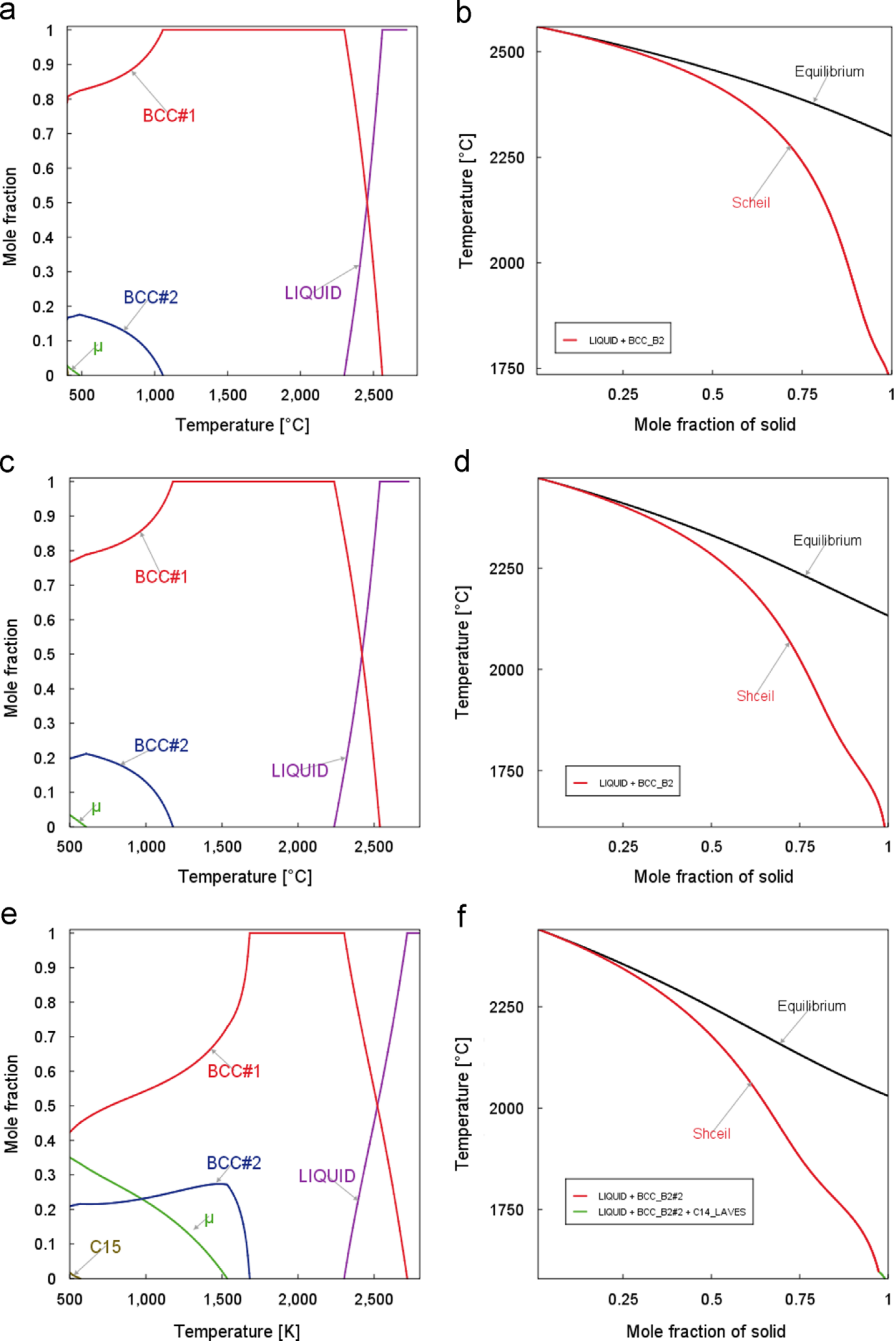
Phase compositions of equilibrium solidification using the measured EDS compositions (a) Cr_0.5_MoNbTaVW, (c) Cr_1.0_MoNbTaVW, (e) Cr_2.0_MoNbTaVW; and Scheil solidification: (b) Cr_0.5_MoNbTaVW, (d) Cr_1.0_MoNbTaVW, (f) Cr_2.0_MoNbTaVW.

The equilibrium solidification process can be simulated by the calculation of the property diagrams. Similar to the site fraction calculations, the POLY3 module was used to calculate the property diagrams with the initial elemental ratios decided by the EDS composition results shown in Fig. 3. Then in the POST module, mole fractions of all the phases are plotted in Fig. 4(a), (c) and (e). The Scheil solidification assumes no diffusion in the solid phase, so the solid phase is excluded from the system in each iteration step of the equilibrium calculation. After the initial conditions for each step were calculated the same way as the equilibrium solidification case, the solid phase amount was fixed to be zero and the temperature was set to be none for the calculation of the liquidus temperature. The liquidius temperature was then used as the new starting point for the next step calculation. In each looping step, only the liquid phase was kept for the equilibrium calculation. After all step calculations were completed in the prescribed temperature range, the mole fractions of the pre-deducted solid phases were plotted in Fig. 4(b), (d) and (f).
